# Supplementary material for: Phenolic Extraction of Moringa oleifera Leaves in DES: Characterization of the Extracts and Their Application in Methylcellulose Films for Food Packaging
Source: Foods. 2022 Aug 31;11(17):2641. doi: 10.3390/foods11172641 (PMC9455762; doi:10.3390/foods11172641)
Supplement: Supplementary file 1 [file foods-11-02641-s001.zip › foods-1869768-supplementary.pdf]

Phenolic extraction of *Moringa oleifera* leaves in DES: characterization of the extracts and their application in methylcellulose films for food packaging

Fazia Braham[1,2], Luísa M.P.F. Amaral[2,3], Krzysztof Biernacki[2], Daniel O. Carvalho[3], Luis F. Guido[3], JúliaM.C.S., Magalhães[2], F. Zaidi[1] , Hiléia K.S. Souza[2,4*] and Maria P. Gonçalves[2]

[1]Département des Sciences Alimentaires, Faculté des Sciences de la Nature et de la Vie, Université de Bejaia, Route Targa Ouzemour, Bejaia 06000, Algeria

[2]REQUIMTE/LAQV, Departamento de Engenharia Química, Faculdade de Engenharia, Universidade do Porto, Rua Dr. Roberto Frias, 4200-465 Porto, Portugal

[3]REQUIMTE/LAQV, Departamento de Química e Bioquímica, Faculdade de Ciências, Universidade do Porto, Rua do Campo Alegre 687, 4169-007 Porto, Portugal

[4] IPC – Institute for Polymers and Composites/I3N, Department of Polymer Engineering, University of Minho, Campus de Azurém, 4800-058 Guimarães, Portugal

* Correspondence: H.K.S. Souza,hileia.souza@dep.uminho.pt

Supplementary Materials:

Table S1: Colour parameters for bread (unwrapped control);

Table S2: Colour parameters for bread wrapped in MC 2% film;

Table S3: Colour parameters for bread wrapped in MC 2% _DES 10% film;

Table S4: Colour parameters for bread wrapped in MC 2% _MO 10% film.

Table S1. Colour parameters for bread (unwrapped control)

Day	L*	a*	b*	a*/b*
0	54.58 ± 3.04 [a]	-0.33 ± 0.09[a]	3.65 ± 0.20	-0.09041
1	56.95 ± 1.92[a]	-0.59 ± 0.02[b]	4.85 ± 0.19	-0.12165
4	58.24 ± 2.62 [b]	-0.60 ± 0.07[b]	5.83 ± 0.33	-0.10292
7	59.93 ± 0.52 [b]	-0.30 ± 0.12 [a]	7.03 ± 0.20 [a]	-0.04267
11	51.04 ± 0.04	0.32 ± 0.01	7.34 ± 0.09 [a]	0.043597

same letters in the same column indicate that values are not significantly different ($p > 0.05$).

Table S2. Colour parameters for bread wrapped in MC 2% film

Day	L*	a*	b*	a*/b*
0				
1	57.06 ± 0.69 [a]	-0.56 ± 0.02 [a]	5.72 ± 0.37 [a]	-0.0979
4	57.99 ± 0.66 [b]	-0.63 ± 0.09 [a]	5.73 ± 0.19 [a]	-0.10995
7	57.32 ± 0.65[a,b]	-0.48 ± 0.02	6.92 ± 0.41	-0.06936
11	50.78 ± 0.13	0.32 ± 0.01	7.54 ± 0.03	0.04244

same letters in the same column indicate that values are not significantly different (p > 0.05).

Table S3. Colour parameters for bread wrapped in MC 2% _DES 10% film

Day	L*	a*	b*	a*/b*
0				
1	57.29 ± 0.501 [a]	-0.07 ± 0.03	5.62 ± 0.13	-0.01246
4	57.90 ± 0.86 [a]	-0.43 ± 0.02	5.93 ± 0.21	-0.07251
7	55.95 ± 0.55 [b]	-0.32 ± 0.03	6.31 ± 0.22	-0.05071
11	55.18 ± 0.02 [b]	0.14 ± 0.02	7.32 ± 0.02	0.019126

same letters in the same column indicate that values are not significantly different (p > 0.05).

Table S4. Colour parameters for bread wrapped in MC 2% _MO 10% film

Day	L*	a*	b*	a*/b*
0				
1	59.02 ± 0.88[a]	-0.40 ± 0.03	6.23 ± 0.27	-0.06421
4	60.93 ± 1.00[a]	-0.62 ± 0.03	5.42 ± 0.12	-0.11439
7	55.98 ± 1.66	-0.32 ± 0.08	6.65 ± 0.20	-0.04812
11	52.83 ± 0.80	0.31 ± 0.01	7.75 ± 0.29	0.04000

same letters in the same column indicate that values are not significantly different (p > 0.05).
